# Apoptosis and necroptosis of mouse hippocampal and parenchymal astrocytes, microglia and neurons caused by *Angiostrongylus cantonensis* infection

**DOI:** 10.1186/s13071-017-2565-y

**Published:** 2017-12-19

**Authors:** Zhang Mengying, Xu Yiyue, Pan Tong, Hu Yue, Yanin Limpanont, Huang Ping, Kamolnetr Okanurak, Wu Yanqi, Paron Dekumyoy, Zhou Hongli, Dorn Watthanakulpanich, Wu Zhongdao, Wang Zhi, Lv Zhiyue

**Affiliations:** 1 Fifth Affiliated Hospital, Zhongshan School of Medicine, Sun Yat-sen University, Guangzhou, 510080 China; 2 Key Laboratory of Tropical Disease Control (Sun Yat-sen University), Ministry of Education, Guangzhou, 510080 China; 3 Provincial Engineering Technology Research Center for Biological Vector Control, Guangzhou, 510080 China; 4 Faculty of Tropical Medicine, Mahidol University, Bangkok, 10400 Thailand; 5 College of Bioscience & Biotechnology, Hunan Agriculture University, Changsha, 410128 China

**Keywords:** *Angiostrongylus cantonensis*, Pathogenesis, Apoptosis, Necroptosis

## Abstract

**Background:**

*Angiostrongylus cantonensis* has been the only parasite among *Angiostrongylidae* to cause human central nervous system infection characterized by eosinophilic meningitis or meningoencephalitis. The mechanism of the extensive neurological impairments of hosts caused by *A. cantonensis* larvae remains unclear. The aim of the present study was to investigate apoptosis, necroptosis and autophagy in the brains of mice infected with *A. cantonensis*, which will be valuable for better understanding the pathogenesis of angiostrongyliasis cantonensis.

**Methods:**

Functional and histological neurological impairments of brain tissues from mice infected with *A. cantonensis* were measured by the Morris water maze test and haematoxylin and eosin (H&E) staining, respectively. The transcriptional and translational levels of apoptosis-, necroptosis- and autophagy-related genes were quantified by quantitative real-time polymerase chain reaction (RT-PCR), and assessed by western blot and immunohistochemistry (IHC) analysis. Apoptotic and necroptotic cells and their distributions in infected brain tissues were analysed by flow cytometry and transmission electron microscopy (TEM).

**Results:**

Inflammatory response in the central nervous system deteriorated as *A. cantonensis* infection evolved, as characterized by abundant inflammatory cell infiltration underneath the meninges, which peaked at 21 days post-infection (dpi). The learning and memory capacities of the mice were significantly decreased at 14 dpi, indicating prominent impairment of their cognitive functions. Compared with those of the control group, the mRNA levels of caspase-3, -4, -6, and RIP3 and the protein levels of caspase-4, cleaved caspase-3, cleaved caspase-6, RIP3, and pRIP3 were obviously elevated. However, no changes in the mRNA or protein levels of FADD, Beclin-1 or LC3B were evident, indicating that apoptosis and necroptosis, but not autophagy, occurred in the brain tissues of mice infected with *A. cantonensis*. The quantitative RT-PCR, western blot, IHC, flow cytometry and TEM results further revealed the apoptotic and necroptotic microglia, astrocytes and neurons in the parenchymal and hippocampal regions of infected mice.

**Conclusions:**

To our knowledge, we showed for the first time that *A. cantonensis* infection causes the apoptosis and necroptosis of microglia and astrocytes in the parenchymal and hippocampal regions of host brain tissues, further demonstrating the pathogenesis of *A. cantonensis* infection and providing potential therapeutic targets for the management of angiostrongyliasis.

## Background

*Angiostrongylus cantonensis* is the most common cause of eosinophilic meningitis worldwide. As accidental hosts, humans can become infected via the ingestion of undercooked intermediate hosts (*Pomacea canaliculata*, *Achatina fulica*, etc.) or contaminated water or food that contains third-stage larvae (L3) [1, 2]. After penetrating the intestinal wall, *A. cantonensis* migrate in the body through the bloodstream, finally passing the blood-brain barrier to further develop in the central nervous system (CNS). Larvae in the brain tissue cause direct mechanical damage and severe inflammation, resulting in eosinophilic meningitis or encephalitis. In addition to the cerebrum and meninges, the cerebellum, brainstem and spinal cord can be affected. Clinical symptoms, manifested as CNS injuries, include severe headache, neck stiffness, convulsions and nausea [3].

Parasitic infections of the CNS include protozoans (*Plasmodium*, *Trypanosoma cruzi*, *Toxoplasma gondii* and amoebae) and metazoans (*Schistosoma*, *Paragonimus*, larval stages of *Taenia solium*, *Echinococcus granulosus*, *Spirometra mansoni*, and *A. cantonensis*), and the pathogenesis of these neuroparasites include the apoptosis and necroptosis of host cells [4]. The major pathogenetic mechanism of cerebral amebiasis lies in the killing of mammalian cells by trophozoite-induced apoptosis [5]. Disruption of the endothelium via apoptosis leads to the leakage of red blood cells, ring haemorrhage, and activated coagulation in adjacent nervous tissue in cerebral malaria [6]. In neurocysticercosis, neurological deficits, particularly learning and memory deficits, are generated by the extensive and significant apoptosis of hippocampal cells [7] and apoptosis of CD3+ T lymphocytes in the brains of hosts induced by *T. solium* cysticerci may be a mechanism by which the parasite downregulates its host’s cellular immune response during early cysticercosis [8]. The most common presentation of cerebral toxoplasmosis in HIV-infected patients is mass lesions consisting of well-defined areas of coagulative necroptosis with or without haemorrhage [9]. The gross appearance of tumoural lesions in neuroschistosomiasis is characterized by the presence of necrotic-exudative granuloma containing eggs surrounded by necrotic and elongated epithelioid cells [10].

Although *A. cantonensis* is the most common cause of eosinophilic meningitis worldwide, the major cell populations and cell injuries in the host brain after infection are not entirely clear [3]. To further reveal the pathogenesis of angiostrongyliasis cantonensis in this study, the Morris water maze test was used to test the neural functionality of *A. cantonensis*-infected hosts. Quantitative RT-PCR, western blot and immunohistochemistry (IHC) analyses were combined to detect apoptosis, necroptosis and autophagy in mouse cerebral parenchymal and hippocampal cells. Flow cytometry and transmission electron microscopy (TEM) were performed to evaluate the cell death of parenchymal and hippocampal astrocytes, neurons and microglia.

## Methods

### Animals

Female BALB/c mice (6–8 weeks old) were purchased from Guangdong Medical Laboratory Animal Center and housed in a specific pathogen-free environment and had unlimited access to food and water.

One batch of mice was randomly divided into 6 groups (9 mice per group) and orally infected with *A. cantonensis* third-stage larvae (L3, 30 per mouse), except for the normal controls. Groups were named according to the number of day(s) post-infection (dpi): group G1 (uninfected mice), group G2 (mice infected for 1 day), group G3 (mice infected for 3 days), group G4 (mice infected for 7 days), group G5 (mice infected for 14 days) and group G6 (mice infected for 21 days). Brain samples from each group were prepared for mRNA and protein extraction or fixed for immunohistochemistry analysis. Animals were euthanized under deep anaesthesia by ether inhalation followed by blood-letting.

Another batch of mice was used for studying neurological function, flow cytometry analysis and transmission electron microscopic observation. They were divided into 4 groups and orally infected with 30 L3 for 7, 14 and 21 days or not infected (control group). After neurological function evaluation, flow cytometry analysis of brain tissues was carried out. Brain tissues from the 0 dpi and 21 dpi groups were subjected to transmission electron microscopy.

### Infecting BALB/c mice with *A. cantonensis* larvae

All the infectious *A. cantonensis* L3 used in this study were obtained from *Biomphalaria glabrata* 21 days after infection of the first-stage larvae (L1) of the parasite. The snails were homogenized and digested in a pepsin-HCl solution (pH 2.0, 500 IU pepsin/g tissue) at 37 °C in an incubator for 40 min. Then, infectious L3 were washed in PBS and counted under an anatomical microscope for animal infection [11].

### Neurological function evaluation

The Morris water maze test was used as described in a previous study [12] to compare learning and memory skills between normal and infected mice. The water maze consisted of a large, circular tank (180 cm in diameter × 60 cm high) filled with water (22 ± 1 °C). A stationary platform was hidden 1.5 cm below the water surface, and white paint was added to make the platform invisible. During cued training, mice were placed into the pool four times each training day (days 1, 2 and 3), once from each of the four quadrants, and then required to find the platform. Animals that located the platform were allowed to rest for 30 s; those that failed to find the platform within 60 s were gently guided to the platform to rest for 30 s. Escape latency, or the time required to reach to the platform, was recorded for each mouse (day 4), and place navigation was used to evaluate their learning and memory skills. The spatial probe test, used to evaluate mouse memory retention, was performed on day 5. The platform was removed, and the probe task was performed in the third quadrant. The swim escape latency, average swim speed, time spent in the target quadrant, and number of times the mice crossed the previous location of the platform were recorded by a video tracking system (Taimeng Tech, Chengdu, China).

### Haematoxylin and eosin (H&E) staining

Mice brains were collected at 0, 1, 3, 7, 14 and 21 dpi, fixed in 4% paraformaldehyde and embedded in paraffin. Brain sections were then de-paraffinized in xylene, rehydrated via graded alcohols and stained with H&E (Biosharp, Wuhan, China) according to the manufacturer’s protocol. Pathological changes were observed under an inverted microscope (Leica, Heidelberg, Germany).

### Sample extraction and cDNA synthesis

Total mouse brain RNA was extracted using TRIzol® Reagent (Thermo Fisher Scientific, Waltham, USA) according to the manufacturer’s protocol, and RNA yield and purity were determined by the NanoDropTM spectrophotometer (Thermo Fisher Scientific). The first cDNA strand was synthesized at 42 °C for 1 h using the RevertAid First Strand cDNA Synthesis Kit (Thermo Fisher Scientific).

### Quantitative real-time polymerase chain reaction (RT-PCR)

For RT-PCR, primers for detecting cell death genes were designed using AlleleID software. The sequences of the target genes and that of the internal control β-actin gene are listed in Table 1. RT-PCR was performed with SYBR® Premix Ex Taq™ (TaKaRa, Dalian, China) in a volume of 20 μl. PCR samples were preheated to 95 °C for 30 s, followed by 40 cycles of 95 °C for 5 s and 60 °C for 20 s. The melt curve was then created by heating at 95 °C for 1 s, 65 °C for 15 s, and 55 °C for 30 s. Reactions were performed on the LightCycler480® Real-Time PCR System (Roche Diagnostics, Reinach, Switzerland), and experiments were repeated three times as independent biological replicates. The mRNA levels of these genes were measured by the Ct value (threshold cycle), and the relative expression levels were calculated with the 2^-ΔΔCt^ method [13].

**Table 1:** The primers used for quantitative real-time PCR

Gene symbol	Forward primer sequence	Reverse primer sequence
Caspase-2	GCACAGGAAATGCAAGAGAA	CTTGGAGCTGAAGCAGTTTG
Caspase-3	AGCAGCTTTGTGTGTGTGATTCTAA	AGTTTCGGCTTTCCAGTCAGAC
Caspase-4	TGTCATCTCTTTGATATATTCCTGAAG	CAAGGTTGCCCGATCAAT
Caspase-6	AGACAAGCTGGACAACGTGACC	CCAGGAGCCATTCACAGTTTCT
Caspase-9	TCCTGGTACATCGAGACCTTG	AAGTCCCTTTCGCAGAAACAG
Cytochrome c	AGGCTGCTGGATTCTCTTACAC	CAGGGATGTACTTTTTGGGATT
Smac	AAGAGCTGCACCAGAAAGCA	TCTGACTGTCAATGGCAGGA
Bcl-2	GTGGATGACTGAGTACCTGAACC	AGCCAGGAGAAATCAAACAGAG
Beclin-1	TGATCCAGGAGCTGGAAGAT	CAAGCGACCCAGTCTGAAAT
Bad	CAGCCACCAACAGTCATC	CTCCTCCATCCCTTCATCC
Bcl-Xl	CTTTCGGGATGGAGTAAAC	AGGTGGTCATTCAGATAGG
RIP3	AAGTGCAGATTGGGAACTACAACTC	AGAATGTTGTGAGCTTCAGGAAGTG
FADD	AAGGTGTCTGGTGGGTGTTC	GCATCAGCAAGAGCAGTAGG
LC3B	CCCCACCAAGATCCCAGT	CGCTCATGTTCACGTGGT
β-actin	GCTGTCCCTGTATGCCTCT	GTCTTTACGGATGTCAACG

### Western blot

Total mouse brain proteins were extracted using Western-IP Lysis Buffer (Beyotime,Wuhan, China) and quantified using the BCA protein assay kit (Beyotime, Wuhan, China). In total, 10 μg of total proteins were electrophoresed on 12% SDS polyacrylamide gels and transferred onto nitrocellulose membranes. The membranes were blocked with PBS/Tween 20® (PBST) containing 5% non-fat milk for 90 min at room temperature and then incubated overnight at 4 °C with the following primary antibodies: anti-caspase-3, anti-cleaved caspase-3, anti-caspase-6, anti-cleaved caspase-6, anti-RIP3, anti-Beclin-1 (Cell Signaling Technology, Danvers, USA), anti-caspase-4, anti-phosphor-RIP3 (Abcam, Cambridge, UK), anti-LC3B (Beyotime,Wuhan, China), and anti-β-actin (Cell Signaling Technology, Danvers, USA) as the control. After incubation with HRP-conjugated goat anti-rabbit IgG or HRP-conjugated goat anti-rat IgG (Cell Signaling Technology), the nitrocellulose membranes were washed three times, and bands were visualized by chemiluminescence with Pierce Enhanced Chemiluminescence Detection Reagent (Millipore, Burlington, USA) on the ChemiDoc™ Touch imaging system (Bio-Rad, Hercules, USA). Relative band intensity was analysed by AlphaViewTM software (Alpha Innotech Corporation, San Jose, USA).

### Immunohistochemistry (IHC)

Mouse brains were isolated, fixed in 4% paraformaldehyde (PFA) overnight at 4 °C and embedded in paraffin. Then, the brain sections were de-paraffinized in xylene, rehydrated via a graded ethanol series, and subjected to antigen retrieval by boiling the slices in citrate buffer (pH 6.0) with high heat for 15 min and medium heat for 15 min in a microwave oven. For IHC analysis, sections were treated with 3% H_2_O_2_ for 10 min to remove endogenous peroxidase, blocked with 1% bovine serum albumin (BSA) in PBS (blocking solution) at room temperature for 1 h, and incubated with anti-caspase-3, anti-cleaved caspase-3, anti-caspase-6 (Cell Signaling Technology), anti-NeuN (Abcam) anti-RIP3 (ABclonal, Wuhan, China), or anti-LC3B (Beyotime) diluted in blocking solution at 4 °C overnight. After being washed 3 times in PBS, the sections were incubated with an HRP-conjugated secondary antibody (DAKO, Glostrup, Denmark) at room temperature for 30 min and then stained with 3, 3′-diaminobenzidine (DAB) for 15 s. Haematoxylin was used for cell nuclei detection. Stained sections were visualized and digitally scanned with an inverted light microscope (Leica).

### Flow cytometry

Mice were euthanized, and their parenchymal and hippocampal tissues were separated, removed and collected into 5 ml of Dounce buffer (Hank’s balanced salt solution containing 15 mM HEPES and 0.5 mM L-glutamine). The samples were homogenized on ice using a glass homogenizer, and the suspensions were pipetted onto a 70 μm cell strainer over a 15 ml conical tube and then centrifuged for 1 min at 1000× *rpm* at 4 °C. The pellets were resuspended in FACS buffer (0.5% BSA, 0.1% NaN_3_ and 25 mM HEPES in PBS) for single-cell suspension preparation. To increase the microglia yield, myelin was removed using Myelin Removal Beads II (Miltenyi Biotec, Bergisch Gladbach, Germany). Cells were blocked using Fc block prior to surface antibody staining at 4 °C for 30 min in the dark. In total, 2.5 μl of the following surface antibodies were used: Annexin V PE-Cyanine7, FVD eFluor 780 (eBioscience, Waltham, USA), CD45-BV421, and CD11b-BV711 (BD Biosciences, Franklin Lakes, USA). After a 20 min incubation, cell suspensions were fixed and permeabilized using fixation/permeabilization buffer (BD Biosciences) and intracellular antibodies (0.5 μl of NeuN-Alexa Fluor 488 and 2.5 μl of PE-conjugated GFAP in 50 μl of BD Perm/WashTM buffer) were then added. Cells were analysed on a CytoFLEX S flow cytometer (Beckman Coulter, Atlanta, USA), and 10,000 cells were collected per tube. Data analysis was performed using FlowJo software (Tree Star, San Carlos, USA).

### Transmission electron microscopy (TEM)

After perfusion with fixatives, small cubes of the latero-ventral hippocampus and parenchymal layers were excised from the brains of normal and 21 dpi mice (*n* = 3 per group) and immediately fixed in 2.5% glutaraldehyde (*v*/v) in 100 mM phosphate buffer with 2% PFA (*w*/*v*) at 4 °C overnight. The cubes were post-fixed in 1% osmium tetroxide in 100 mM phosphate buffer for 1.5 h at room temperature. The samples were then washed, dehydrated in a graded series of ethanol (50, 70, 80, 90 and 100%), cleared in acetone and embedded in Eponate 12 resin (Ted Pella Inc., Redding, USA). Ultrathin sections (60 nm thickness) were cut with a diamond knife (Diatome, Nidau, Switzerland) on a Leica EM UC7 ultra-microtome, stained with a 2.0% uranyl acetate solution and a 2.0% lead citrate solution, and observed using a Tecnai G2 Spirit Twin electron microscope (FEI, Hillsboro, USA) operated at 80 kV.

### Statistical analyses

Statistical analyses were performed using GraphPad Prism 5.0 (GraphPad Software, San Diego, USA) with two-tailed unpaired Student’s t-test and SPSS 20.0 (IBM Corp., NY, USA) with one-way analysis of variance (ANOVA) followed by the Tukey-Kramer test. The data are presented as the means ± standard deviation (SD). Differences among comparisons were considered statistically significant for *P*-values less than 0.05.

## Results

### Morris water maze test

**Fig. 1:**
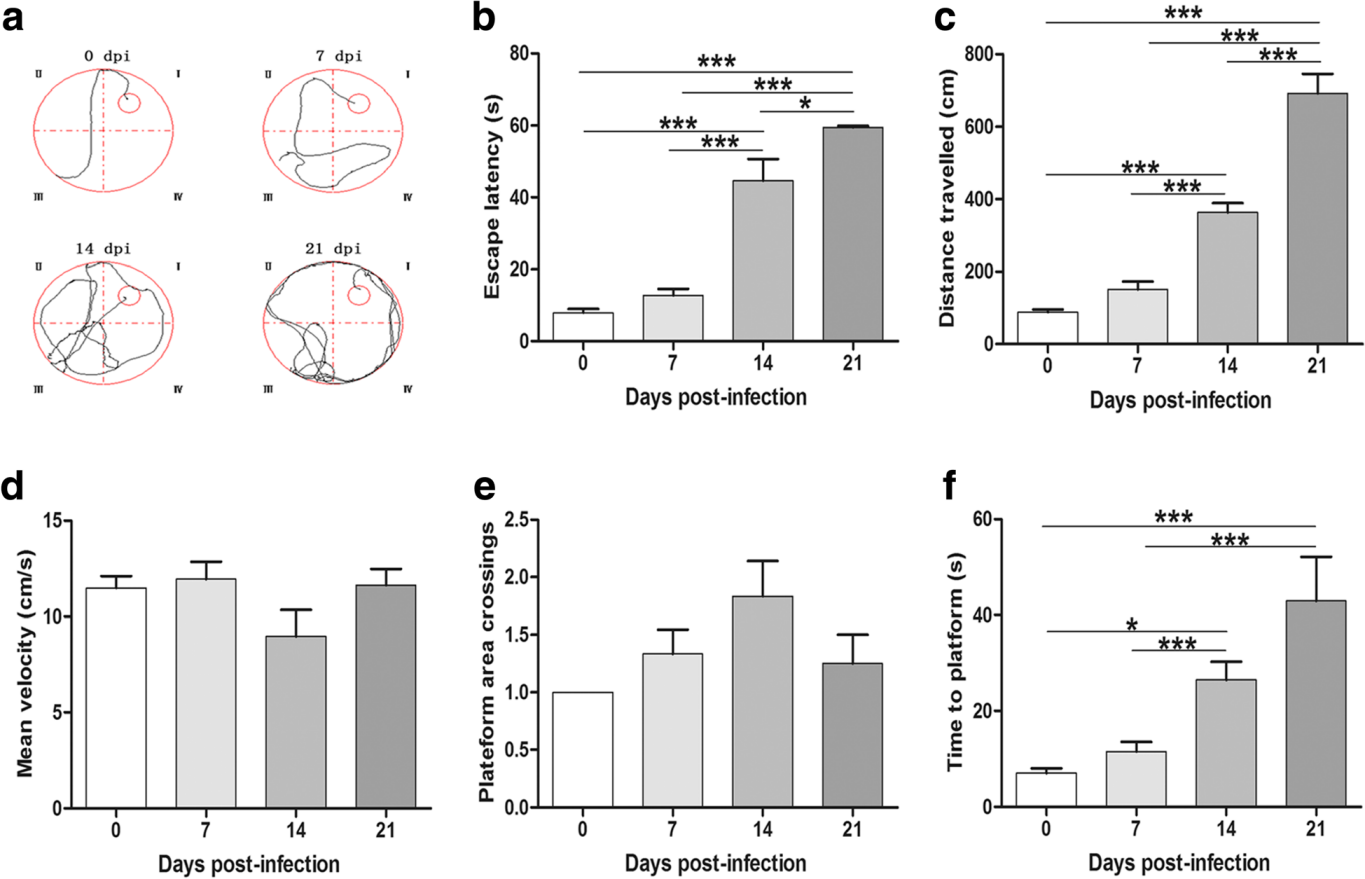
Infected mice exhibited impaired spatial learning and memory skills. **a**-**d** Results of the place navigation test: the moving tracks (**a**), the latency to reach platform (**b**), the travelled distance before reaching the platform (**c**) and the average swimming speed of the mice in different groups during place navigation test) (**d**). **e**, **f** Results of spatial probe test: the number of target location crossings of the mice during the probe trial (**e**) and the time spent for the mice to reach the platform during probe trial in which platform was removed (**f**). Values are expressed as means ± SD (*n* = 6). *P < 0.05, ***P < 0.001

During the 4 day place navigation test, significantly (*F*_(3,20)_ = 60.19, *P* < 0.0001 and *F*_(3,20)_ = 73.31, *P* < 0.0001, respectively) prolonged escape latency times and extended swimming distances were observed in the 14 dpi (44.65 ± 14.87 s and 362.89 ± 63.82 cm, respectively) and 21 dpi (59.5 ± 1.04 s and 692.49 ± 130.11 cm, respectively) groups compared with mice in the 0 dpi (7.93 ± 2.51 s and 88.31 ± 17.99 cm, respectively) and 7 dpi (12.76 ± 4.35 s and 150.86 ± 50.48 cm, respectively) groups. In addition, disparity in the above two parameters between the 14 dpi and 21 dpi groups was statistically significant (*F*_(3,20)_ = 60.19, *P* = 0.019 and *F*_(3,20)_ = 73.31, *P* < 0.0001, respectively) (Fig. 1a-c), while no significant differences in average swimming speed were observed between normal and infected mice (*P >* 0.05) (Fig. 1d). In the spatial probe test on day 5, no significant distinction among the groups in terms of platform area crossing were observed (*P* > 0.05) (Fig. 1e). However, mice in the 14 dpi (26.47 ± 9.37 s) and 21 dpi (42.93 ± 18.34 s) post-infection groups significantly extended their time spent in the target quadrant compared with the 0 dpi (6.97 ± 2.48 s) and 7 dpi (11.48 ± 5 s) groups (*F*_(3,20)_ = 14.25, *P* < 0.0001), while no significant difference in the time spent in the target quadrant between the 21 dpi and 14 dpi groups was observed (*P* > 0.05) (Fig. 1f). The results indicated a clear time-dependent reduction in the learning and memory retention capacities of mice infected with *A. cantonensis*.

### Histopathological observation of the brains of mice infected with *A. cantonensis*

**Fig. 2:**
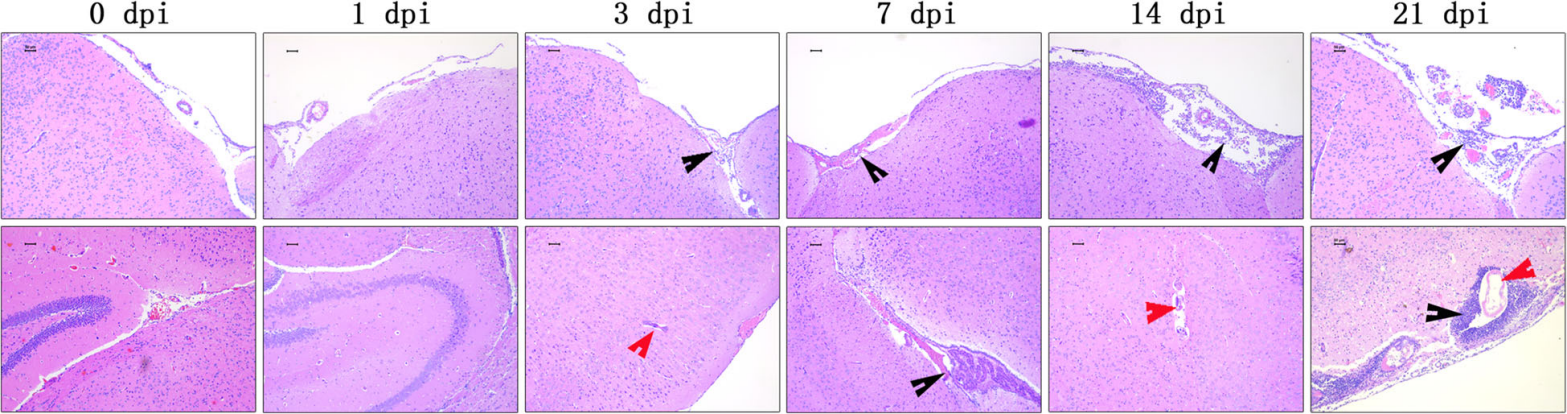
Histopathological injuries of mice infected with *A. cantonensis*. The black arrows show that inflammatory cells infiltrated into the brain of the infected mouse, and aggregated on the meninges and caused thickening of the meninges. The red arrows indicate the worms in the parenchyma of brains of the infected mice. (100× magnification). *Scale-bars*: 50 μm

H&E staining reflected the histopathological injuries and inflammation of mouse brains infected with *A. cantonensis*. No histopathological injuries or inflammatory cells were observed in the brains of the 0 dpi and 1 dpi groups. After 3 dpi, a few inflammatory cells infiltrated into the mouse brains, aggregating on and thickening the meninges (black arrows). Worm transection in the infected parenchyma illustrated that this parasite could move into the brain after only 3 days of infection (red arrows). As the infection time extended, more inflammatory cells infiltrated into the mouse brains, and their histopathological statuses became more serious (Fig. 2).

### Detection of mRNA levels by quantitative RT-PCR

**Fig. 3:**
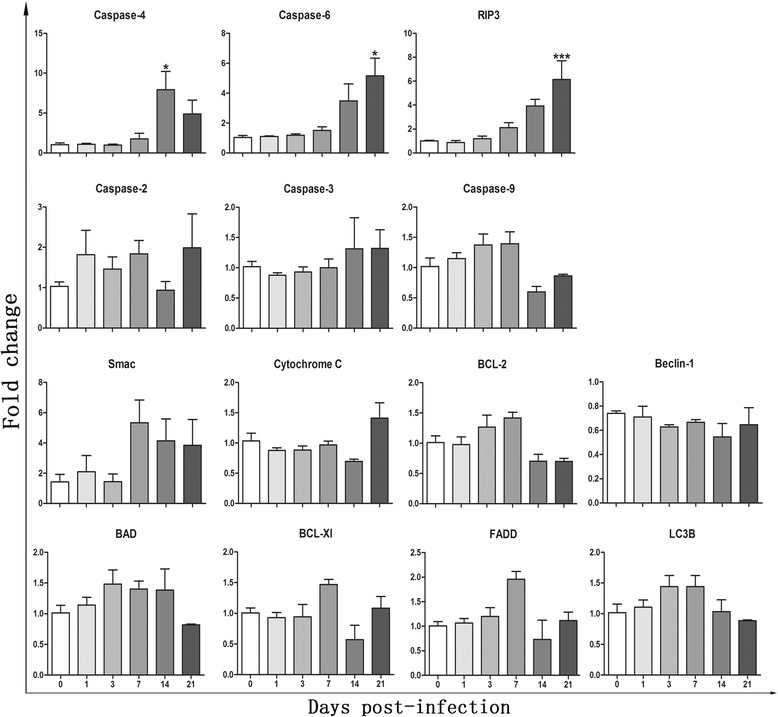
Messenger RNA levels of cytokines related to apoptosis, necroptosis and autophage in brains of mice were detected by RT-qPCR at different time points (0, 1, 3, 7, 14 and 21 dpi) (*n* = 3). Values are expressed as means ± SD. *P < 0.05 *vs* 0 dpi, **P < 0.01 *vs* 0 dpi, ***P < 0.001 *vs* 0 dpi

To detect brain injuries in the infected mice, the mRNA levels of some molecules related to apoptosis, necroptosis and autophagy, including caspase-2, -3, -4, -6, -9, Cc, Smac, Bcl-2, Bad, Bcl-Xl, RIP3, FADD, Beclin-1 and LC3B, were measured. The mRNA levels of caspase-4, -6 and RIP3 in the infected mouse brains were 7-, 5- and 6-fold higher than those of normal mice. The mRNA levels of caspase-4, -6 and RIP3 increased as brain inflammation progressed, and the caspase-6 and RIP3 mRNA levels peaked and had statistical significance at 21 dpi (*F*_(5,12)_ = 6.232, *P* = 0.0004 and *F*_(5,12)_ = 8.755, *P <* 0.0001, respectively). The mRNA level of caspase-4 peaked at 14 dpi (*F*_(5,12)_ = 6.386, *P* = 0.0051) (Fig. 3).

### Determination of protein expression using western blot

**Fig. 4:**
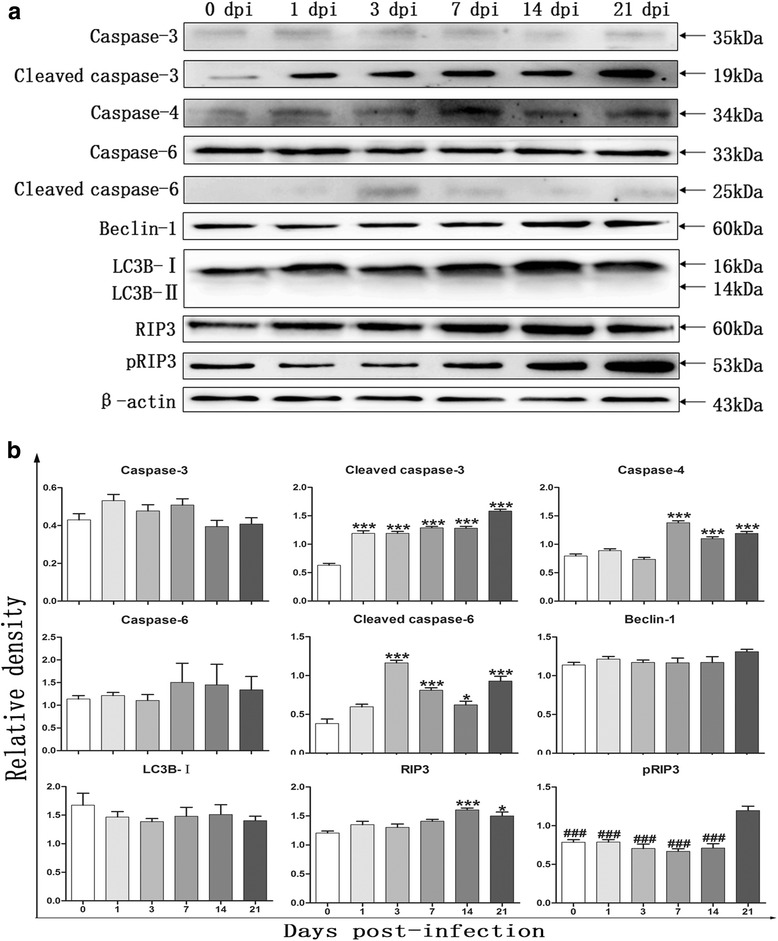
**a** Expression levels of proteins associated with apoptosis, necroptosis and autophage were determined by western blot in different groups (0, 1, 3, 7, 14 and 21 dpi). **b** Densitometric analysis (mean ± SD, *n*   =  3) of caspase-3, -4, -6, cleaved caspase-3, -6, Beclin-1, LC3B-I, RIP3 and phospho-RIP3 normalized to the endogenous control and expressed as fold change. *P < 0.05 and ***P < 0.001, compared with 0 dpi group; ^###^ *P* < 0.001, compared with 21 dpi group

We next detected the expression levels of proteins associated with apoptosis, necroptosis and autophagy by western blot. Figure 4 shows obviously increased levels of the apoptotic proteins cleaved caspase-3 (*F*_(5,12)_ = 80.86, *P* < 0.0001), caspase-4 (*F*_(5,12)_ = 56.65, *P* < 0.0001) and cleaved caspase-6 (*F*_(5,12)_ = 35.58, *P* < 0.0001) after *A. cantonensis* infection, and these levels peaked at 21 dpi, 7 dpi and 3 dpi, respectively. RIP3 expression was clearly upregulated from 14 dpi (*F*_(5,12)_ = 8.458, *P* = 0.0009) to 21 dpi (*F*_(5,12)_ = 8.458, *P* = 0.011) and peaked at 14 dpi. pRIPK3, an active mediator of necroptosis, was significantly elevated at 21 dpi (*F*_(5,12)_ = 17.17, *P <* 0.0001). However, there was no significant effect on the expression levels of total caspase-3, total caspase-6 and Beclin-1 proteins compared to those of normal mice, and no clear accumulation of LC3B-II can be observed in the mice infected with the parasite (*P* > 0.05) (Fig. 4).

### Caspase-3 and RIP3 in parenchymal and hippocampal cells

**Fig. 5:**
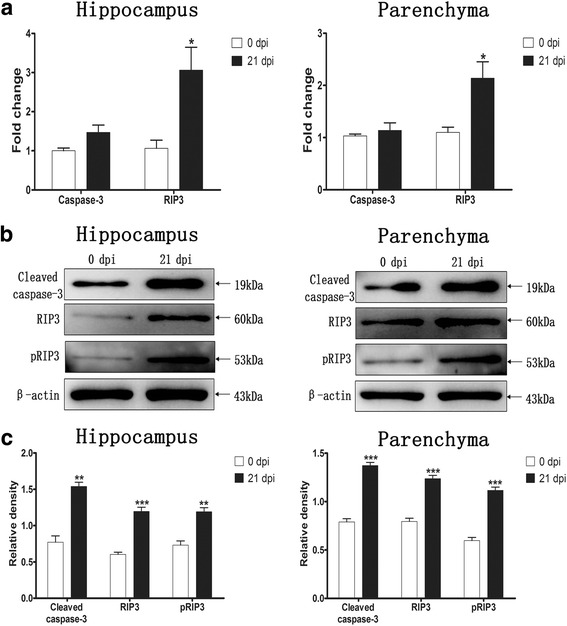
RT-qPCR and western blot analyses of caspase-3, cleaved caspase-3, RIP3 and pRIP3 in parenchymal and hippocampal cells (*n* = 3). **a** RT-qPCR products obtained with the total RNA of uninfected mice (0 dpi) and mice at 21 dpi with caspase-3- or RIP3-specific primers. **b** The expression levels of cleaved caspase-3, RIP3 and pRIP3 in mouse hippocampal and parenchymal cells were confirmed by western blot. **c** Densitometric analysis of cleaved caspase-3, RIP3 and pRIP3 normalized to the endogenous control (β-actin) and expressed as fold change. The values are expressed as the mean ± SD. *P < 0.05, **P < 0.01, ***P < 0.001 compared with the 0 dpi group

Caspase-3 and RIP3 expression in the parenchymal and hippocampal cells of the 0 dpi and 21 dpi groups were analysed by quantitative RT-PCR and western blot. A significant elevation in the RIP3 mRNA level after infection was observed, as it was 2- and 3-fold higher than those of normal mice in parenchymal (*t*_(4)_ = 4.826, *P* = 0.017) and hippocampal cells (*t*_(4)_ = 3.624, *P* = 0.0152), respectively. Furthermore, no obvious alterations of the caspase-3 mRNA level were observed (*P >* 0.05) (Fig. 5a). Western blot analysis showed a correspondingly significant elevation of RIP3 and pRIP3 expression in post-infectious parenchymal (*t*_(4)_ = 9.374, *P* = 0.0007 and *t*_(4)_ = 11.03, *P* = 0.0004, respectively) and hippocampal cells (*t*_(4)_ = 9.139, *P* = 0.0008 and *t*_(4)_ = 5.619, *P* = 0.0049, respectively). Similar conclusions were found regarding the cleaved caspase-3 protein levels in parenchymal cells (*t*_(4)_ = 12.35, *P* = 0.0002) and hippocampal cells (*t*_(4)_ = 7.3, *P* = 0.0019) (Fig. 5b, c). All data indicated that *A. cantonensis* infection could result in apoptosis and necroptosis in mouse hippocampal and parenchymal cells.

### Apoptosis and necroptosis in infected mouse parenchymal and hippocampal cells as detected by immunohistochemistry (IHC)

**Fig. 6:**
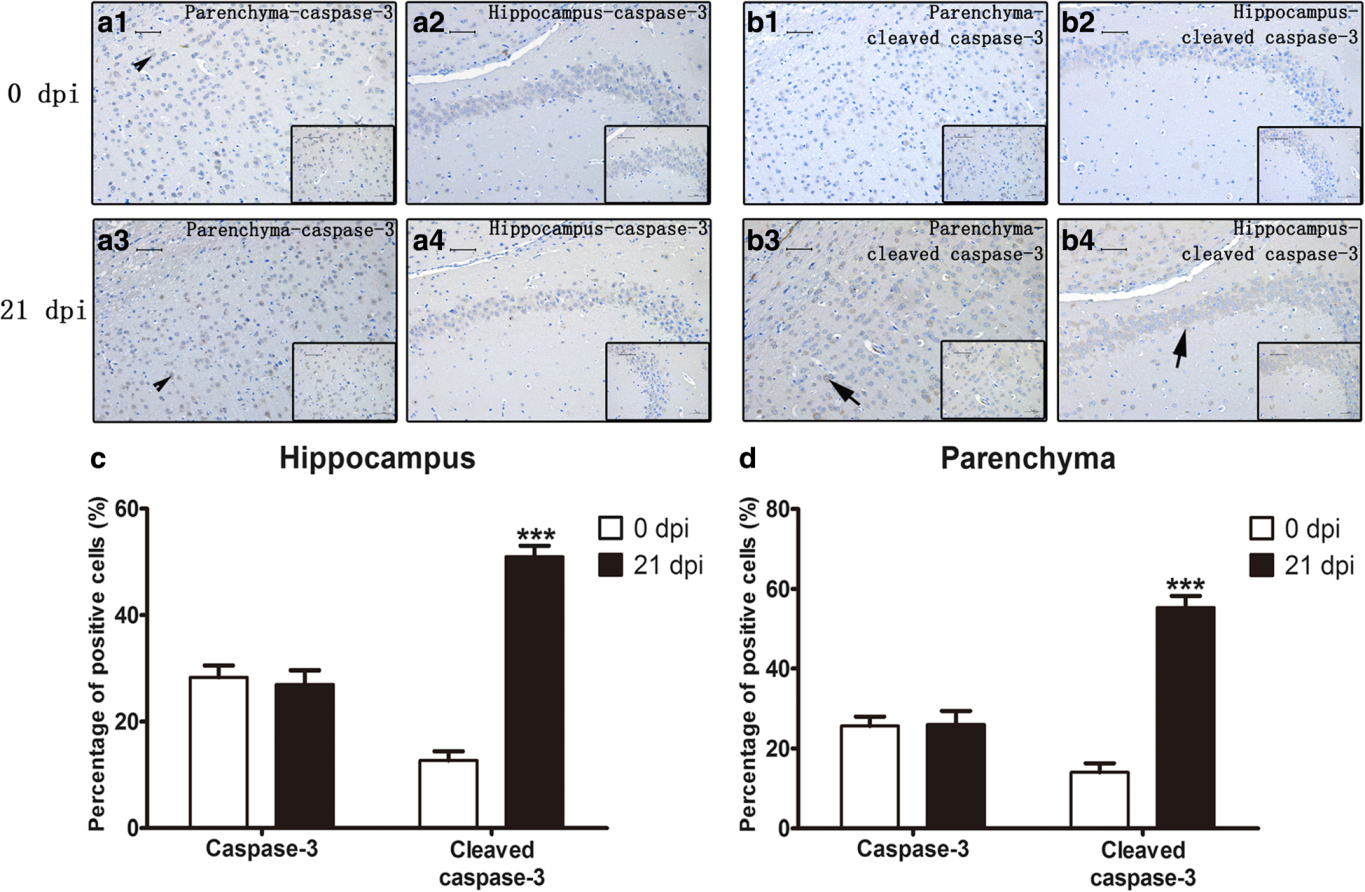
Immunohistochemistry (IHC) staining of parenchymal and hippocampal cells in 0 dpi and 21 dpi groups to visualize the expression and localization of caspase-3 and cleaved caspase-3. The expression levels of parenchymal caspase-3 (**a1**) and hippocampal caspase-3 (**a2**) in the 0 dpi group, parenchymal caspase-3 (**a3**) and hippocampal caspase-3 (**a4**) in the 21 dpi group, parenchymal cleaved caspase-3 (**b1**) and hippocampal cleaved caspase-3 (**b2**) in the 0 dpi group, and parenchymal cleaved caspase-3 (**b3**) and hippocampal cleaved caspase-3 (**b4**) in the 21 dpi group were measured by IHC. Arrowheads and arrows indicate caspase-3-positive cells and cleaved caspase-3-positive cells, respectively (magnification: 200×; insets: 400×). **c**, **d** The percentages of positive cells in the hippocampus (**c**) and parenchyma (**d**). *Scale-bars*: 50 μm; insets: 50 μm

**Fig. 7:**
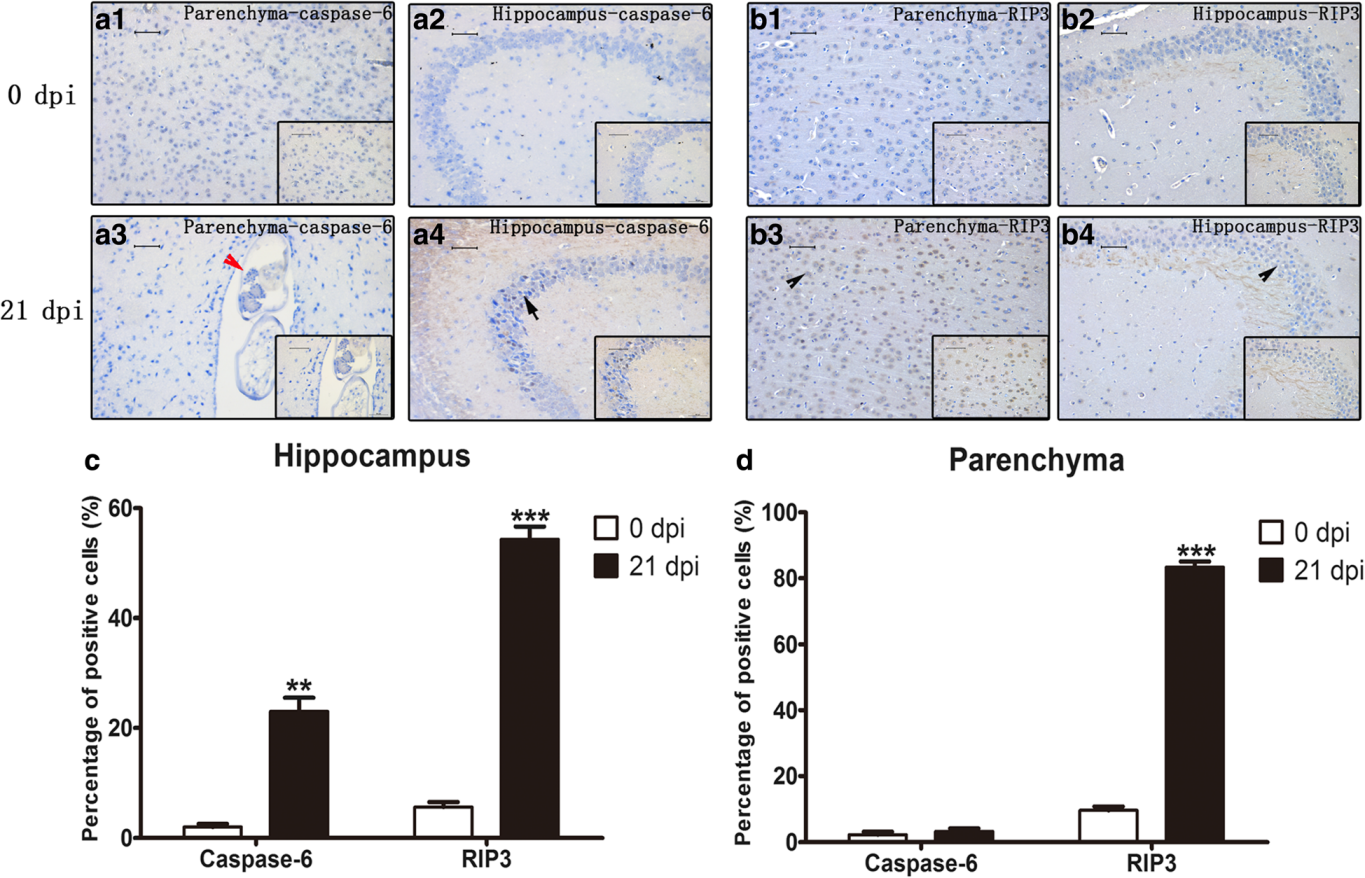
IHC staining of parenchymal and hippocampal cells in 0 dpi and 21 dpi groups to visualize the expression and localization of caspase-6 and RIP3. **a1** Parenchymal caspase-6 expression in the 0 dpi group. **a2** Hippocampal caspase-6 expression in the 0 dpi group. **a3** Parenchymal caspase-6 expression in the 21 dpi group. **a4** Hippocampal caspase-6 expression in the 21 dpi group. **b1** Parenchymal RIP3 expression in the 0 dpi group. **b2** Hippocampal RIP3 expression in the 0 dpi group. **b3** Parenchymal RIP3 expression in the 21 dpi group. **b4** Hippocampal RIP3 expression in the 21 dpi group. Black arrowhead indicates caspase-6-positive cells; arrow indicates RIP3-positive cells; red arrowhead indicates the worms in parenchyma of the mouse brain (magnification: 200×; insets: 400×). **c** Percentage of caspase-6- and RIP3-positive cells in the hippocampus. **d** Percentage of caspase-6- and RIP3-positive cells in parenchyma. *Scale-bars*: 50 μm; insets: 50 μm

**Fig. 8:**
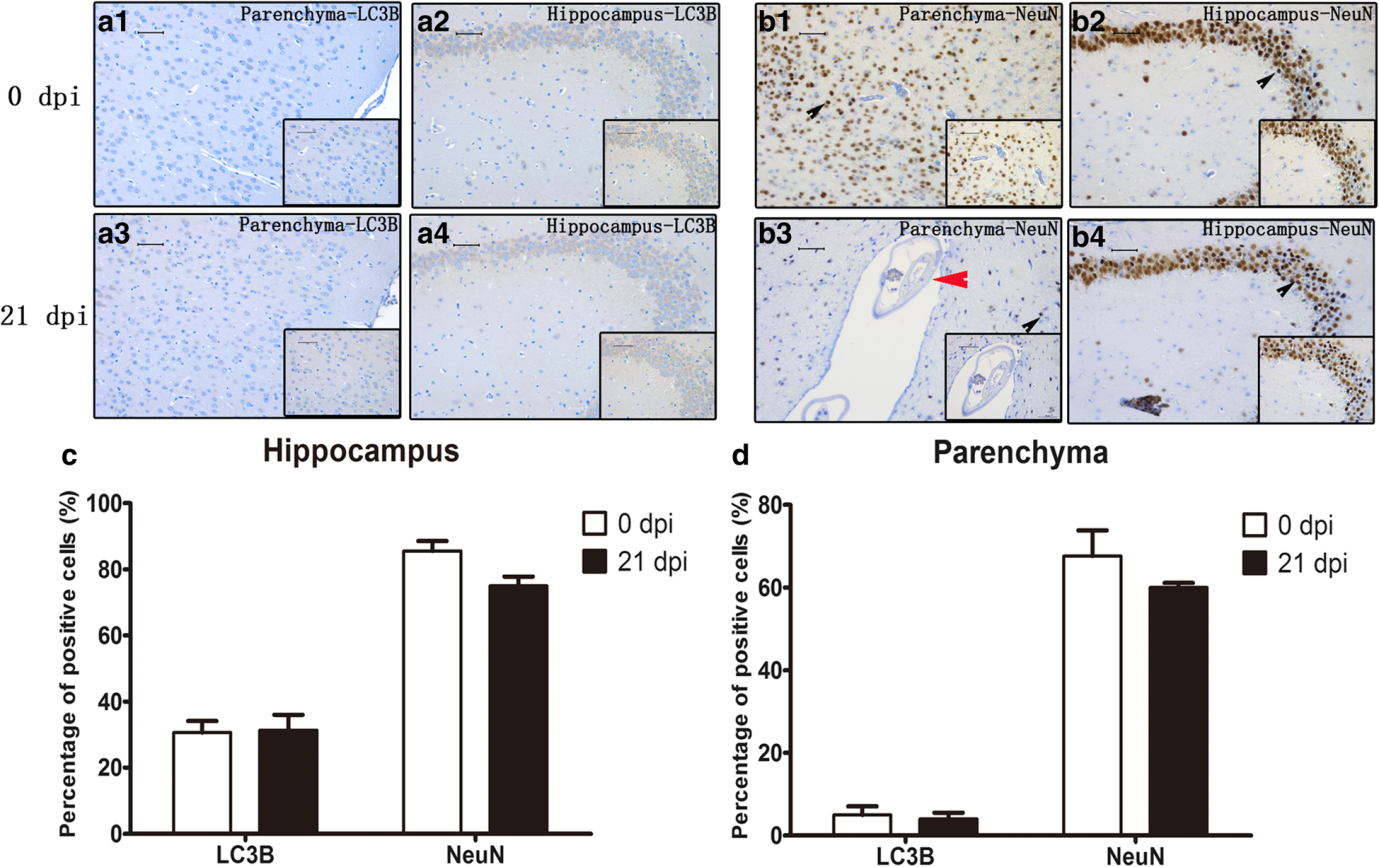
IHC staining of parenchymal and hippocampal cells in the 0 dpi and 21 dpi groups to visualize the expression and localization of LC3B and NeuN. **a1** Parenchymal LC3B expression in the 0 dpi group. **a2** Hippocampal LC3B expression in the 0 dpi group. **a3** Parenchymal LC3B expression in the 21 dpi group. **a4** Hippocampal LC3B expression in the 21 dpi group. **b1** Parenchymal NeuN expression in the 0 dpi group. **b2** Hippocampal NeuN expression in the 0 dpi group. **b3** Parenchymal NeuN expression in the 21 dpi group. **b4** Hippocampal NeuN expression in the 21 dpi group. Black arrowhead indicates neurons, red arrowhead indicates worms in parenchyma of the mouse brain (magnification: 200×; insets: 400×). **c** Percentage of LC3B- and NeuN-positive cells in the hippocampus. **d** Percentage of LC3B- and NeuN-positive cells in parenchyma. *Scale-bars*: 50 μm; insets: 50 μm

**Fig. 9:**
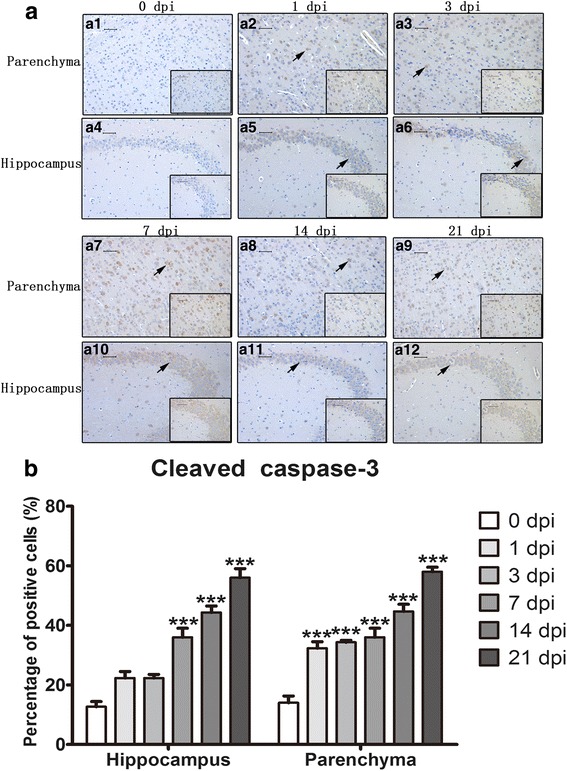
IHC staining of brain parenchymal and hippocampal cells in the 0 dpi group and at various post-infectious stages to compare the expression and localization of cleaved caspase-3. **a1** Parenchyma, 0 d,pi . **a2** Parenchyma, 1 dpi. **a3** Parenchyma, 3 dpi. **a4** Hippocampus, 0 dpi. **a5** Hippocampus, 1 dpi. **a6** Hippocampus, 3 dpi. **a7** Parenchyma, 7 dpi. **a8** Parenchyma, 14 dpi. **a9** Parenchyma, 21 dpi. **a10** Hippocampus, 7 dpi. **a11** Hippocampus, 14 dpi. **a12** Hippocampus, 21 dpi. Arrow indicates cleaved caspase-3-positive cells (magnification 400×). **b** Percentage of cleaved caspase-3-positive cells in hippocampal and parenchymal tissue. *Scale-bars*: 50 μm; insets: 50 μm

**Fig. 10:**
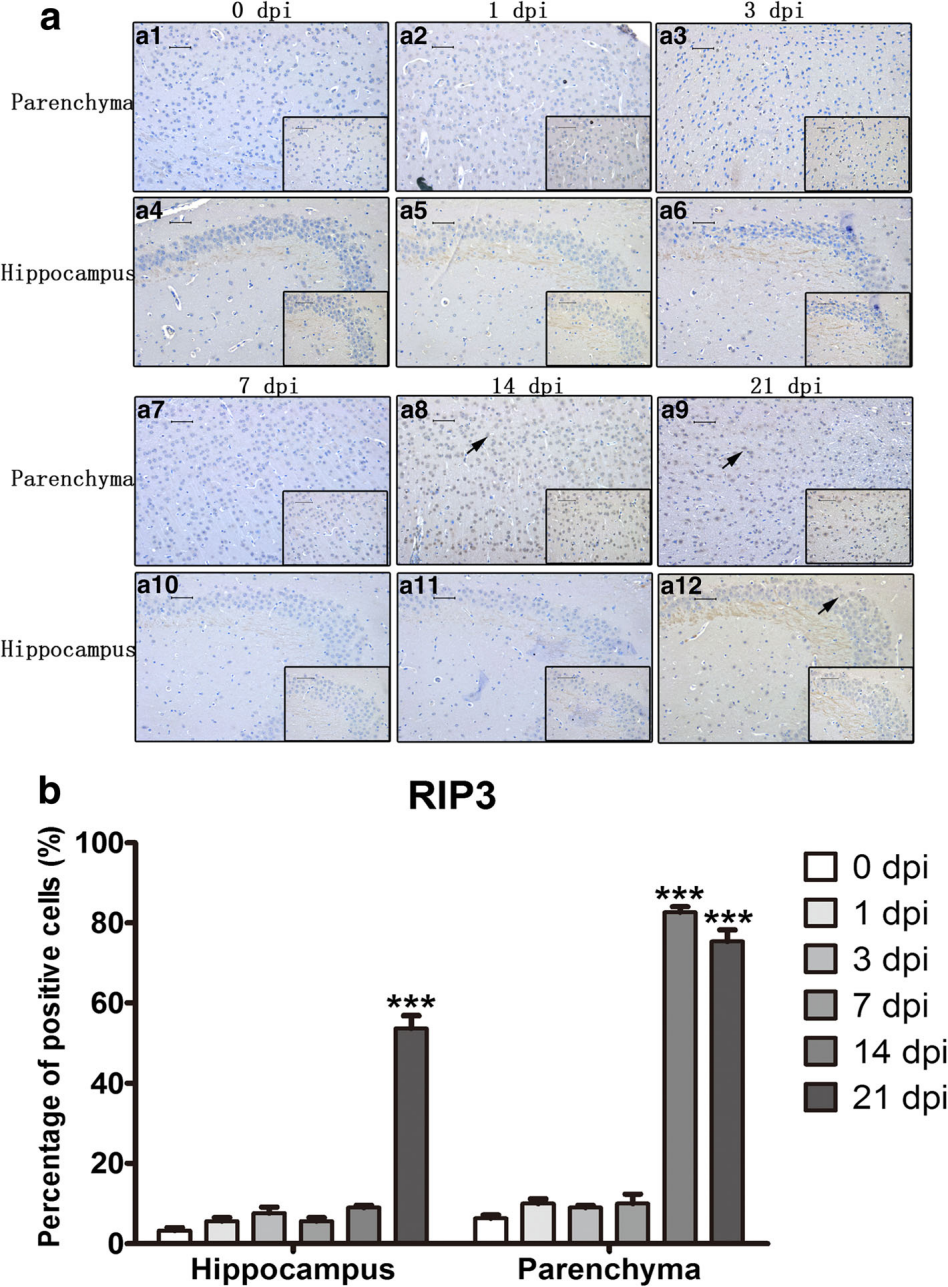
IHC staining of brain parenchymal and hippocampal cells in the 0 dpi group and at various post-infectious stages to compare the expression and localization of RIP3. **a1** Parenchyma, 0 dpi. **a2** Parenchyma,1 dpi. **a3** Parenchyma, 3 dpi. **a4** 0 dpi hippocampus. **a5** Hippocampus, 1 dpi. **a6** Hippocampus, 3 dpi. **a7** Parenchyma, 7 dpi. **a8** Parenchyma,14 dpi. **a9** Parenchyma, 21 dpi. **a10** Hippocampus, 7 dpi. **a11** Hippocampus, 14 dpi. **a10** Hippocampus, 21 dpi. Arrow indicates RIP3-positive cells (magnification 200×; insets: 400×). **b** Percentage of RIP3-positive cells in hippocampal and parenchymal cells. *Scale-bars*: 50 μm; insets: 50 μm

Expression and localization of caspase-3, cleaved caspase-3, caspase-6, RIP3, LC3B and NeuN were detected in the 0 dpi and 21 dpi groups by IHC. As shown in Figs. 6, 7, 8, cleaved caspase-3- and RIP3-positive cells were significantly increased in both parenchymal and hippocampal cells of the 21 dpi group. For cleaved caspase-3, the percentage of positive cells increased from 12.67 ± 3.06% to 51 ± 3.61% and 14 ± 4% to 55.33 ± 5.03% in the hippocampus and parenchyma, respectively. The number of RIP3-positive cells increased from 5.67 ± 1.53% to 54.33 ± 4.04% and 9.67 ± 2.08% to 88.33 ± 3.06% in the hippocampus and parenchyma, respectively. A mild elevation in the number of caspase-6-positive cells from 2 ± 1% to 23 ± 4.36% was observed in infected hippocampi, and no substantial changes were observed in the number of caspase-3-positive cells. Neither normal nor infected mouse brains exhibited LC3B-positive cells, implying that *A. cantonensis* infection does not lead to autophagy. Different infectious stages were then measured by similar procedures. The number of cleaved caspase-3-positive cells gradually multiplied from 1 dpi to 21 dpi and peaked at 58 ± 2.65% in parenchymal cells and 56 ± 5.29% in hippocampal cells at 21 dpi (Figs. 9 and 10). Parenchymal RIP3-positive cells peaked at 14 dpi at 82.67 ± 2.31%, while hippocampal RIP3-positive cells peaked at 21 dpi at 53.67 ± 5.51% (Figs. 9 and 10). The IHC results further proved that post-infectious apoptosis and necroptosis occur in mouse hippocampal and parenchymal cells.

**Fig. 11:**
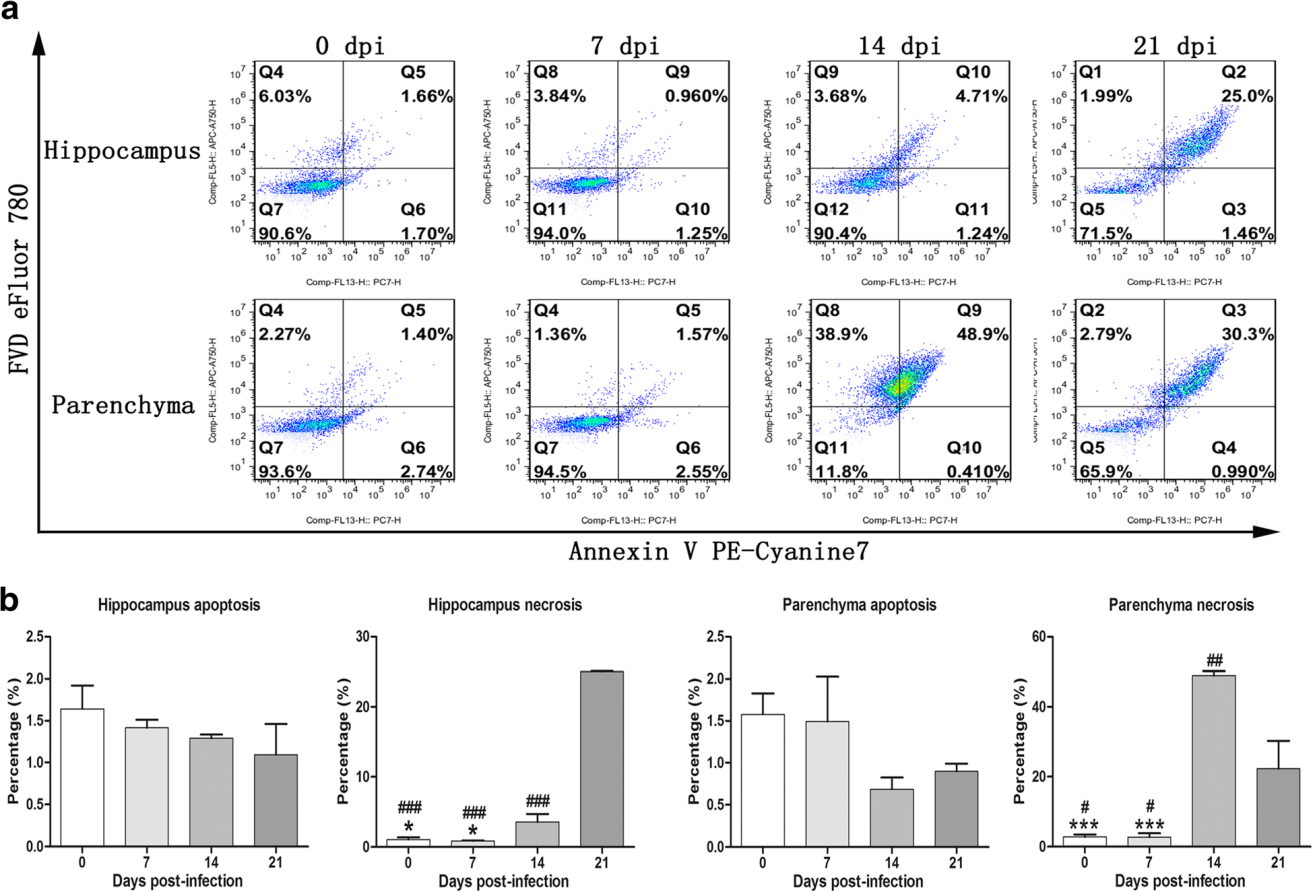
**a** Flow cytometry analysis of parenchymal and hippocampal cell death caused by *A. cantonensis* infection (*n* = 3). **b** The graph shows the means of three independent experiments that were performed in triplicate. The values are expressed as the mean ± SD. *P < 0.05 and ***P < 0.001 *vs* the 14 dpi group; ^#^ *P <* 0.05, ^##^ *P <* 0.01 and ^###^ *P <* 0.001 *vs* the 21 dpi group

**Fig. 12:**
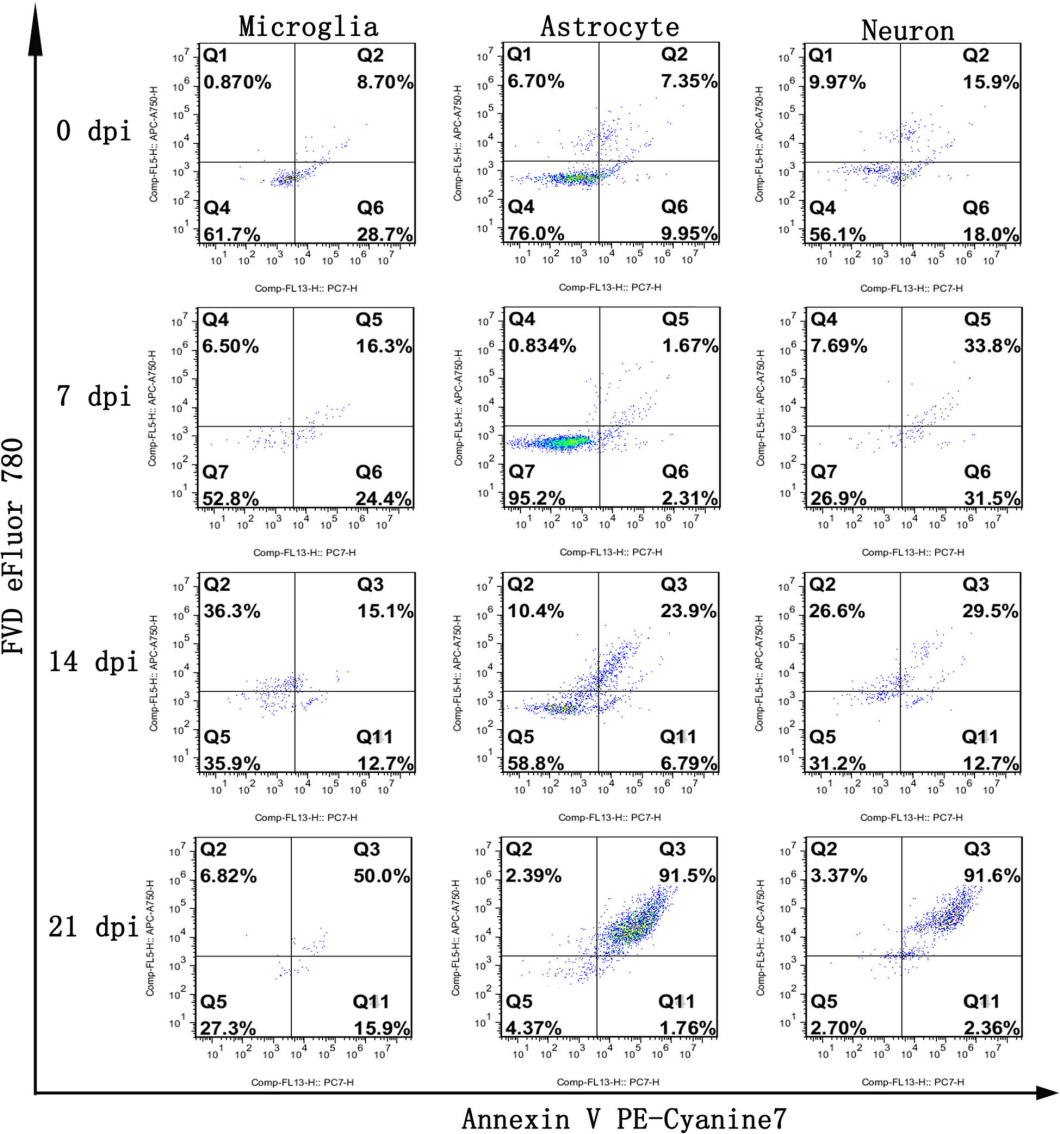
Effect of *A. cantonensis* infection on mouse hippocampal cell death as analysed by flow cytometry

**Fig. 13:**
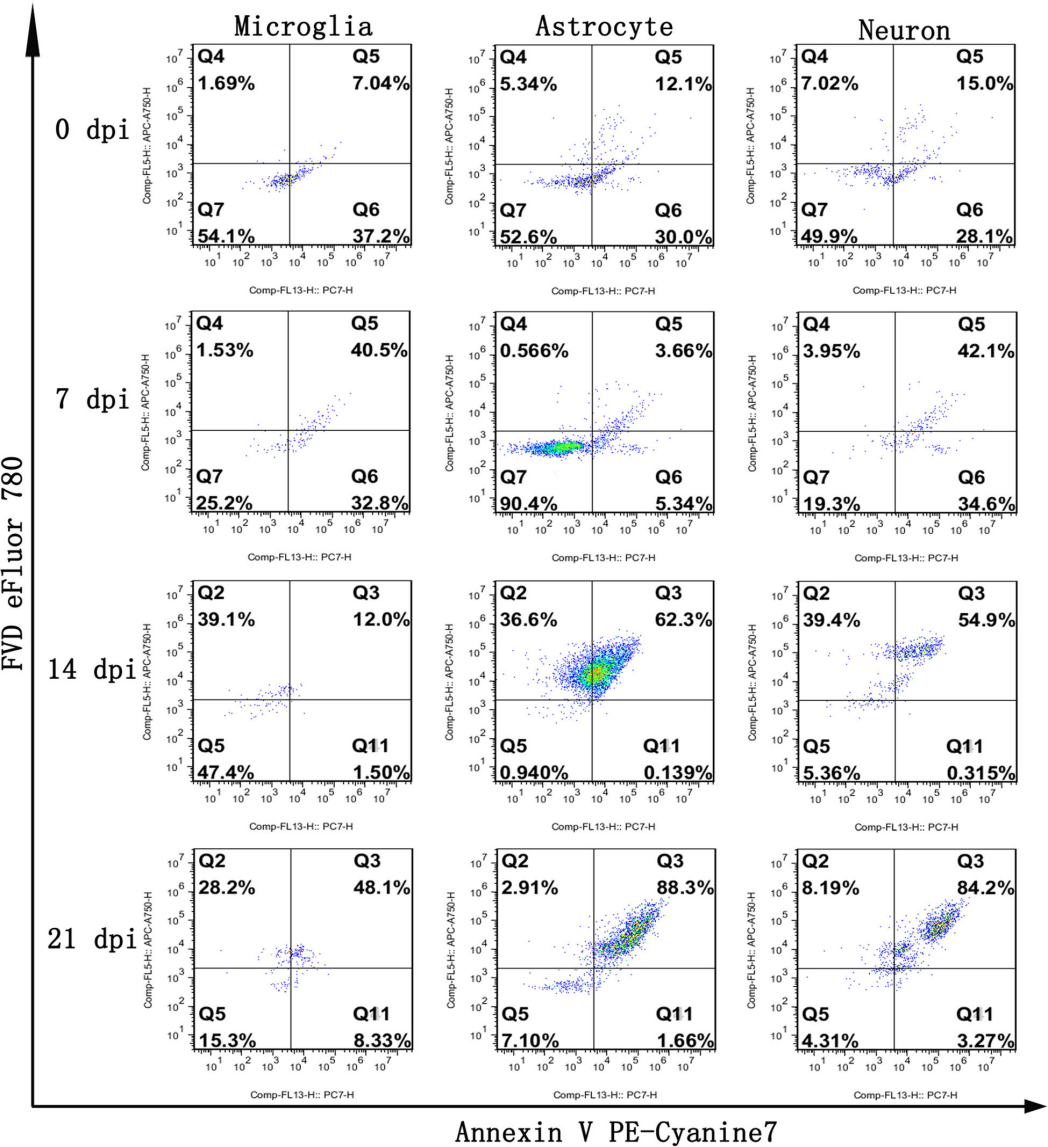
Effect of *A. cantonensis* infection on mouse parenchymal cell death as detected by flow cytometry

**Fig. 14:**
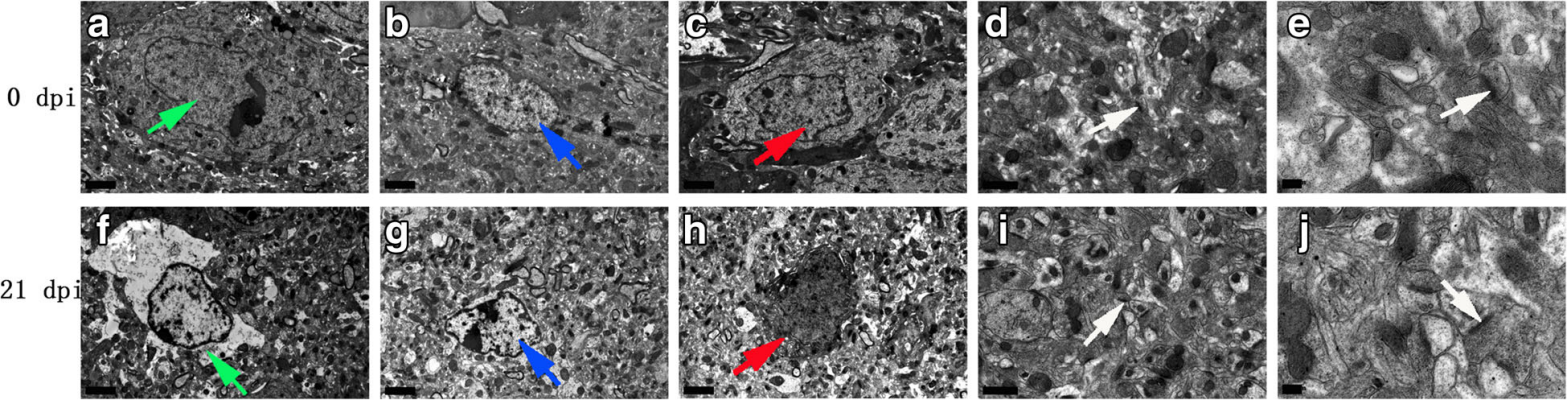
Representative transmission electron micrographs of mouse hippocampal cells in the 0 dpi (**a**-**e**) and 21 dpi (**f**-**j**) groups. The green, blue, red and white arrows indicate astrocytes, microglia, neurons and intercellular synapses, respectively. **a**-**c** Normal appearance and ultrastructure. **f** Swollen and ruptured astrocytes. **g** Swollen microglia. **h** Shrunken neurons. No obvious synapse lesions were observed in **d**, **e**, **i**, **j** (magnification: **a**-**c**, **f**-**h**, 5800×; **d**, **i**, 13,500×; **e**, **j**, 37,000×). *Scale-bars*: **a**-**c**, **f**-**h**, 2 μm; **d**, **i**, 1 μm; **e**, **j**, 200 nm

**Fig. 15:**
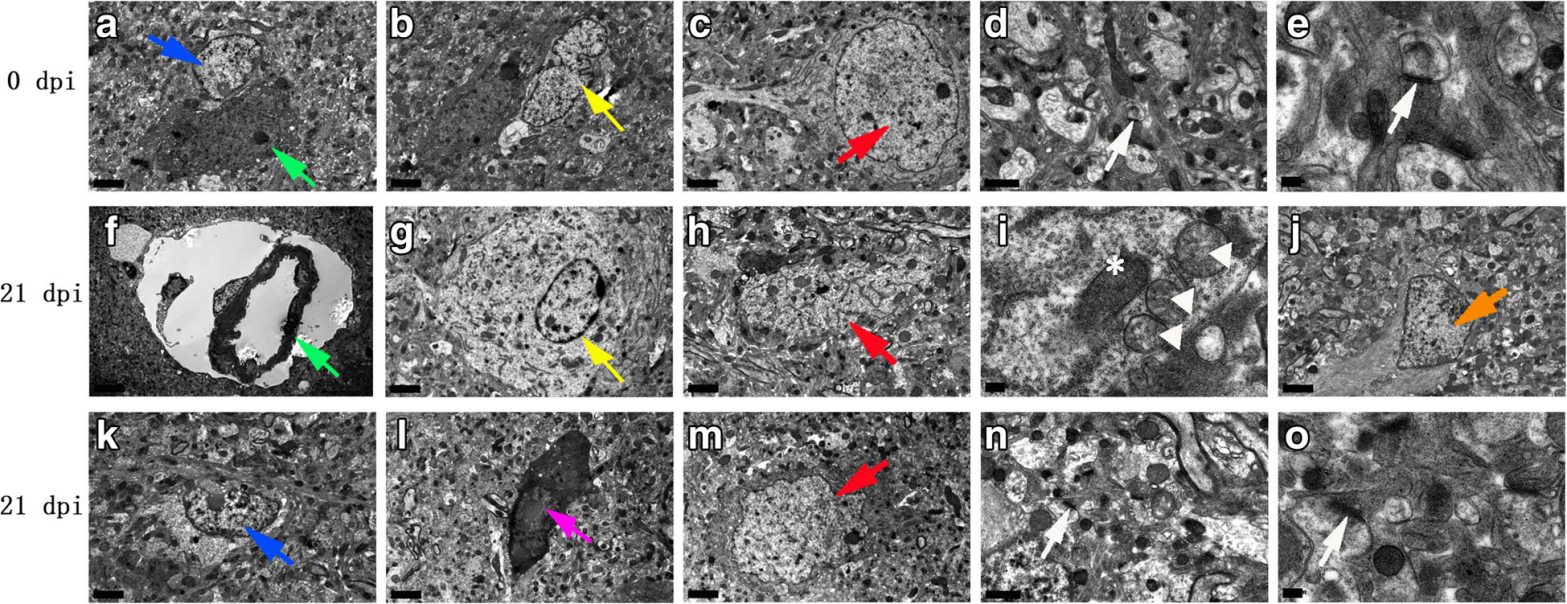
Representative transmission electron micrographs of mouse parenchymal cells in the 0 dpi (**a**-**e**) and 21 dpi (**f**-**o**) groups. The green, blue, yellow, red and white arrows indicate astrocytes, microglia, oligodendrocytes, neurons and intercellular synapses, respectively. **a**-**c** Normal appearance and ultrastructure. **f**, **k** Swollen astrocytes and microglia, respectively. **g** Swollen oligodendrocytes. **h**, **m** Swollen and shrunken neurons, respectively. **i** Organelle alterations in swollen neurons (asterisk indicates normal mitochondria; triangle indicates swollen mitochondria). **j** Abnormally emerged smooth muscle cells (orange arrow). **l** Apoptotic cells (purple arrow). No obvious synapse lesions were observed in **d**, **e**, **n**, **o** (magnification: **a**-**c**, **f**-**h**, **j**-**m**: 5800×; **d**, **n**, 13,500×; **e**, **i**, **o**: 37,000×). *Scale-bars*: **a**-**c**, **f**-**h**, **j**-**m**, 2 μm; **d**, **n**, 1 μm; **e**, **i**, **o**, 200 nm

### Detection of cell death by flow cytometry

Distinction between early-apoptotic cells and late-apoptotic/necrotic cells was detected by flow cytometry using the Annexin V PE-Cyanine7 and FVD eFluor 780 double-label staining technique. Proportions of late-apoptotic or necrotic parenchymal cells (Annexin V PE-Cyanine7+/FVD eFluor 780+) in the 14 dpi (48.9 ± 2.3%) and 21 dpi (22.25 ± 11.38%) groups were significantly increased compared with those in the 0 dpi (2.81 ± 1.27%) and 7 dpi (2.79 ± 1.88%) groups (Fig. 11). Notably, the late-apoptotic or necrotic cell counts peaked at 14 dpi (*F*_(3,8)_ = 66.33, *P <* 0.0001), followed by a slight decline at 21 dpi in parenchymal cells. The latter still showed statistically significant distinction between the 0 dpi/7 dpi (*F*_(3,8)_ = 66.33, *P* = 0.01) and 14 dpi groups (*F*_(3,8)_ = 66.33, *P =* 0.002) compared with 21 dpi. Similarly, the proportions of late-apoptotic or necrotic hippocampal cells in the 14 dpi (3.58 ± 1.58%) and 21 dpi (25.05 ± 0.07%) groups were also significantly increased compared with those in the 0 dpi (1.09 ± 0.49%) (*F*_(3,8)_ = 590.3, *P* = 0.032 and *F*_(3,8)_ = 590.3, *P* < 0.0001, respectively) and 7 dpi (0.90 ± 0.06%) group (*F*_(3,8)_ = 590.3, *P* = 0.023 and *F*_(3,8)_ = 590.3, *P* < 0.0001, respectively). The late-apoptotic and necrotic cell counts in hippocampal cells peaked at 21 dpi, with a statistically significant difference from those of the 14 dpi group (*F*_(3,8)_ = 590.3, *P <* 0.0001). Furthermore, no significant distinctions in early-apoptotic cell counts (Annexin V PE-Cyanine7+/FVD eFluor 780-) were found in any of the groups. The flow cytometry results revealed that apoptosis and necroptosis occur in hippocampal and parenchymal cells during *A. cantonensis* infection. The apoptotic and necrotic cell categories were further evaluated using different surface biomarkers (CD45 and CD11b for microglia, NeuN for neurons, GFAP for astrocytes). As indicated in Figs. 12 and 13, as the infection time extended, distinct elevations in the proportions of parenchymal and hippocampal astrocytes, neurons and microglia undergoing late apoptosis or necroptosis were observed, and almost 90% of the astrocytes and neurons had underwent apoptosis and necroptosis at 21 dpi.

### Morphological changes in mouse brains infected with *A. cantonensis* as observed by TEM

Compared with the normal morphology and ultrastructure of cells in the 0 dpi group, the astrocytes (Figs. 14, 15f), microglia (Figs. 14g, 15k), oligodendrocytes (Fig. 15g) and neurons (Fig. 15h) of the 21 dpi group showed apparent swelling, rupture and loss of nuclear membrane integrity and visible swollen organelles (Fig. 15i), indicating that these cells underwent necrosis. Neuronal pyknosis and shrinkage (Figs. 14h, 15m) revealed that the neurons may have undergone apoptosis. Characteristics of apoptotic cells, such as patchy nuclear membranes, chromatin margination (Fig. 15l) and abnormally emerged smooth muscle cells (Fig. 15j), implied apoptosis and necroptosis of parenchymal and hippocampal cells. Hence, we concluded that significant morphological changes in hippocampal and parenchymal cells occur after *A. cantonensis* infection.

## Discussion

Eosinophilic meningitis or eosinophilic meningoencephalitis in humans is commonly caused by *Angiostrongylus cantonensis*, a food-borne zoonotic parasite, in southern Asia and the Pacific and Caribbean islands [14, 15]. The clinical symptoms of angiostrongyliasis cantonensis are characterized by severe headache, neck stiffness, paraesthesias and cranial nerve palsy [16, 17]. The neurological manifestations of neuroangiostrongyliasis include eosinophilic meningitis, encephalitis/encephalomyelitis, radiculitis and cranial nerve abnormalities, ataxia, tremors and paralysis, which have been observed in previous clinical reports [18–20] and in studies on infected experimental animal models [11, 21]. These characteristics coincide with our results that infected mice exhibited extensive brain injury and neurological disorder, as detected by the Morris water maze test (Fig. 1) and H&E staining (Fig. 2). However, the pathogenesis of *A. cantonensis* neurological dysfunction remains poorly understood. The tracks and microcavities caused by the migration of larvae in the brains of infected animals, as described in previous studies [21], cannot explain all the clinical neurological injuries in the hosts [20] or the distinct outcomes between permissive and non-permissive hosts after *A. cantonensis* infection since the same physical destruction of neural tissue caused by the migration of larvae can be seen within both of their brains [11]. Interestingly, our previous [22] and the current findings indicate the obvious apoptosis and necroptosis of parenchymal and hippocampal astrocytes, neurons and microglia of mice infected with *A. cantonensis* (Figs. 3–10), which might provide new insights into the pathogenesis of neuroangiostrongyliasis.

The cell is the basic structural and functional unit of life. Cell death, as a natural process of life, plays an important role in the development and homeostatic balance of multicellular organisms [23–25]. According to morphology and molecular mechanisms, cell death is mainly divided into three categories: apoptosis, autophagy, and necroptosis (or necrotic cell death) [26]. Apoptosis is an automatic and programmed process regulated by signalling pathways, including the death receptor signalling, mitochondria-mediated and endoplasmic reticulum stress pathways [27, 28]. All the pathways ultimately activate caspases to complete apoptosis. Caspase-3, -4, and -6 are important members of the caspase family [29], as their function in orchestrating apoptosis has been widely confirmed [30]. In this study, we discovered that compared with those of uninfected mice, the caspase-4 and -6 mRNA levels and cleaved caspase-3, -4, and -6 expression levels of *A. cantonensis*-infected mice were elevated. As shown in Figs. 3 and 4, *A. cantonensis* infection could cause the apoptosis of mouse brain cells. Furthermore, qPCR, western blot, immunohistochemistry, flow cytometry and TEM analysis further proved that apoptosis mainly occurs in parenchymal and hippocampal astrocytes, neurons and microglia during *A. cantonensis* infection (Figs. 5–15). Although previous studies showed an elevation in the number of apoptotic leukocytes in post-infective parenchyma and subarachnoid space and the induction of apoptosis in endothelial cells and astrocytes after the addition of larvae extracts [31, 32], we observed for the first time in this study that *A. cantonensis* infection caused significant apoptosis amongst parenchymal and hippocampal astrocytes, neurons and microglia, which could partially account for the severe neural damage observed (Fig. 1). In addition, we first confirmed that the levels of RIP3 and pRIP3 increased as the infection time extended (Figs. 3, 4). RIP3 is the key to apoptosis and necroptosis and a specific marker for necroptosis [33]. Thus, the increased RIP3 and pRIP3 levels indicated that *A. cantonensis* infection could induce the necroptosis of astrocytes, neurons and microglia (Figs. 5–15). Necroptosis is a recently recognized mechanism of programmed cell death that, unlike apoptosis, shows necrotic morphological changes with a patterned regulatory mechanism [34, 35]. Necroptosis can be induced by TNF-α, the activator of programmed cell death [36–38], and is mediated by its unique RIP3-dependent signalling pathway. In most cases, apoptosis and necroptosis co-exist, and a main difference between them is that necroptosis is accompanied by inflammation [39], causing inflammatory diseases, such as neurodegenerative disorders, haemorrhage-induced brain damage, Crohn’s disease, multiple sclerosis and certain viral infections [40]. Our study on *A. cantonensis* infection-induced brain apoptosis and necroptosis is not only consistent with our previous work in that the level of TNF-α, a trigger of apoptosis and necroptosis that is increased in the brains of mice infected with *A. cantonensis* [41], can be directly induced by the cystatin from *A. cantonensis* (unpublished data), but also further explains the pathogenesis of severe neural dysfunction accompanied with extensive eosinophilic CNS inflammation during *A. cantonensis* infection.

As the macrophages of the brain, microglia play both protective and toxic (damaging) roles in non-infectious pathological states, such as brain damage or neurodegenerative disorders [42, 43]. As the only immune cells in cerebral parenchyma, microglia are considered the first-line barrier protection for the CNS against pathogens (bacteria, viruses, parasites, etc.) [44–46]. Astrocytes are the most widely distributed cells in the mammalian brain and the largest population among glial cells [47]. Thus, astrocytes play significant roles in survival functionality, neuronal development and information transmission, neuronal maturation and synapse formation, CNS metabolism, and damaged blood-brain barrier repair and reconstruction [48, 49]. Neurons are the basic structural and functional units of the nervous system, and the key to neural function is the synaptic signalling process, which is partly electrical and partly chemical [50]. The essential function of neurons is to exchange messages through information input, integration, conduction and output. Thus, neurons are crucial to the sensation, learning, memory, cognition and movement of organisms [51, 52]. The apoptosis or necroptosis of microglia, astrocytes or neurons can lead to severe neural dysfunction (epilepsy, Alzheimer’s disease, cerebrovascular disease, encephalitis, meningitis, etc.), or even death [53, 54]. Apoptosis and necroptosis occurred in microglia, astrocytes or neurons after *A. cantonensis* infection, indicating that neurotrophic agents and inhibitors of apoptosis and necroptosis could be effective in combination with anti-parasitic drugs (albendazole) and steroidal anti-inflammatory drugs (dexamethasone).

## Conclusions

In summary, this study is the first to demonstrate that *A. cantonensis* infection can significantly induce the apoptosis and necroptosis of astrocytes, neurons and microglia in host brain parenchymal and hippocampal tissues. This not only enriches our understanding of *A. cantonensis* pathogenesis but also provides a clue to novel potential therapeutic strategies against angiostrongyliasis cantonensis.

## Abbreviations

dpi: Days post-infection
HE: Hematoxylin and eosin
IHC: Immunohistochemical
L1: First-stage larvae
L3: Third-stage larvae
RT-PCR: Real-time polymerase chain reaction
TEM: Transmission electron microscopy
